# Assessment of Factors Associated with Unfavorable Outcomes among Drug-Resistant TB Patients: A 6-Year Retrospective Study from Pakistan

**DOI:** 10.3390/ijerph19031574

**Published:** 2022-01-29

**Authors:** Farman Ullah Khan, Asim ur Rehman, Faiz Ullah Khan, Khezar Hayat, Amjad Khan, Nafees Ahmad, Jie Chang, Usman Rashid Malik, Yu Fang

**Affiliations:** 1 Department of Pharmacy Administration and Clinical Pharmacy, Xi’an Jiaotong University, Xi’an 710061, China; farman.khan@abasynisb.edu.pk (F.U.K.); fkhan@bs.qau.edu.pk (F.U.K.); khezar.hayat@uvas.edu.pk (K.H.); jiechang@xjtu.edu.cn (J.C.); usmanmalik_ucp@hotmail.com (U.R.M.); 2 Center for Drug Safety and Policy Research, Xi’an Jiaotong University, Xi’an 710061, China; 3 Shaanxi Center for Health Reform and Development Research, Xi’an Jiaotong University, Xi’an 710061, China; 4 Research Institute for Drug Safety and Monitoring, Institute of Pharmaceutical Science and Technology, China’s Western Technological Innovation Harbor, Xi’an 710061, China; 5 Department of Pharmacy, Faculty of Biological Sciences, Quaid-i-Azam University, Islamabad 45320, Pakistan; arehman@qau.edu.pk (A.u.R.); amjadkhan@qau.edu.pk (A.K.); 6 Institute of Pharmaceutical Sciences, University of Veterinary and Animal Sciences, Lahore 54000, Pakistan; 7 Department of Pharmacy Practice, Faculty of Pharmacy and Health Sciences, University of Baluchistan Quetta, Quetta 08769, Pakistan; nafeesuob@gmail.com

**Keywords:** drug-resistant tuberculosis, antibiotics, epidemiology, factors, treatment success

## Abstract

The spread of drug-resistant tuberculosis (DR TB) poses significant challenges to the control and successful eradication of TB globally. The current retrospective study was designed to evaluate the treatment outcomes and identify the risk factors associated with unsuccessful outcomes among DR TB patients. A total of 277/308 eligible DR TB patients were enrolled for treatment at the programmatic management unit of DR TB at the Pakistan Institute of Medical Sciences, Islamabad between January 2014 and July 2019. Treatment outcomes were defined according to the WHO recommendations. Death, treatment failure, and lost to follow-up (LTFU) were collectively grouped as unsuccessful treatment outcomes, whereas cured and treatment completed were summed up together as successful treatment outcomes. Out of the total 277 patients, 265 (95.67%) were multidrug/rifampicin-resistant TB (MDR/RR-TB) cases, 8 (2.89%) were isoniazid resistant cases, and 4 (1.44%) were extensively drug-resistant ones. In the current cohort, a total of 177 (63.9%) achieved successful treatment outcomes. Among them, 153 (55.2%) were declared cured and 24 (8.7%) completed their treatment. Of the remaining 100 (36.1%) patients with unsuccessful outcomes, 60 (21.7%) died, 32 (11.5%) were LTFU, and 8 (2.9%) had failed treatment. The proportion of male patients was relatively higher (55.2%), within the age group of 21–40 years (47.3%) and lived in rural areas (66.8%). The multivariate analysis revealed that unsuccessful outcomes had a statistically significant association with being male (adjusted odds ratio, AOR: 1.92, 95% confidence interval (CI): 1.10–3.36), being in an age group above 60 years (AOR: 3.34, 95% CI: 1.09–10.1), suffering from any comorbidity (AOR: 2.69, 95% CI: 1.35–5.38), and the history of use of second-line drugs (AOR; 3.51, 95% CI 1.35–9.12). In conclusion, treatment outcomes among DR TB patients at the study site were poor and did not achieve the treatment success target (≥75%) set by the World Health Organization.

## 1. Introduction

Irrespective of global efforts, tuberculosis (TB) continues to be a leading public health concern [1]. The spread of drug-resistant tuberculosis (DR TB) remains a threat to the TB control. Approximately half a million cases occurred worldwide in 2019 [2]. Treatment regimens used against DR TB are costly, prolonged, less effective, and are associated with more side effects as compared with drug-susceptible TB [3]. As a result, the global treatment success rate for DR TB remains less than 60%, and a large number of patients die each year [4,5]. The World Health Organization (WHO) states that approximately 9% of DR TB patients have a more likely chance of unsuccessful outcomes and their disease further develop into extensively drug-resistant TB (XDR TB) [6]. Currently, less developed countries are confronted with many DR TB cases that are alarmingly increasing every year [7]. To optimize the DR TB care and prevention requires a thorough understanding of the main factors that lead to poor treatment outcomes.

According to the WHO, Pakistan ranks fifth in the Eastern Mediterranean Region for DR TB [8,9]. The country has come a long way in enhancing DR TB management through several initiatives, such as the establishment of direct observation short course therapy (DOTs) and programmatic management of DR TB. Despite these efforts in the past decades, the country continues to face significant challenges in controlling and eradicating DR TB [1,10]. According to a study, the incidence of new drug-resistant TB cases was 4%, whereas 19.4% were previously TB treated patients [11]. Similarly, A. Javaid et al., in 2018, reported that mortality due to DR cases is increasing every year in Pakistan [5]. To improve the successful outcomes of DR TB, there must be a consistency with the DOTs rules related to the management [12]. To maintain a consistency in management, it is essential to identify socioeconomic factors at the population level that create hurdles in care and prevention [1]. Therefore, the WHO has directed that the treatment outcomes for DR TB patients must be regularly reviewed at national and district levels [13]. Regular monitoring of treatment outcomes will not only support to assess the performance of national TB program, but it will also help to identify treatment sites that require improvement in the future. Nevertheless, there have been no reports of DR TB patients’ treatment outcomes and socioeconomic factors at the population level from the study site. Therefore, the current study was designed to investigate treatment outcomes and risk factors associated with unsuccessful outcomes among drug-resistant TB patients in Pakistan.

## 2. Materials and Methods

### 2.1. Study Design

The current retrospective observational study was carried out at the Programmatic Management of Drug-resistant Tuberculosis (PMDT) unit at the Pakistan Institute of Medical Sciences, Islamabad, Pakistan (PIMS). The study center is well-equipped and has a staff that includes doctors, nurses, data operators, coordinators, pharmacists, psychologists, and other supporting staff. All culture-confirmed drug-resistant TB patients enrolled for treatment at the study site between January 2014 and July 2019 were included in the final analysis. It covers patients from different parts of the country, primarily registered from the capital (Islamabad), and two provinces of Pakistan, namely Punjab and Khyber Pakhtunkhwa, and a self-governing state, Azad Jammu and Kashmir.

### 2.2. Study Data Collection and Eligibility Criteria

A standardized data collection form based on WHO guidelines for the management of DR TB, previously published studies, and recommendations of the supervisory committee and healthcare professionals at the study site was used to abstract patients’ sociodemographic, microbiological, and clinical data from Electronic Nominal Record System records (ENRS) and patients’ medical record files. Enrolled patients were retrospectively followed until their end treatment outcomes were reported. Patients were carefully examined by specialist clinicians, data coordinators, and pharmacists to manage care.

**Figure 1:**
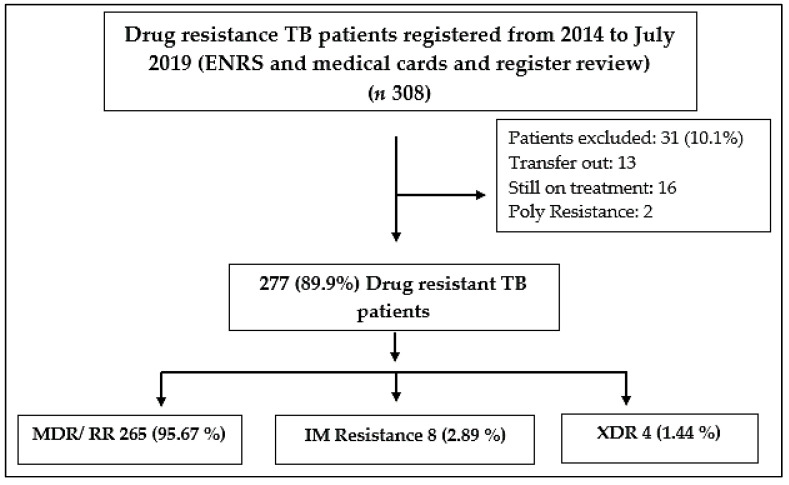
Overall drug resistance patient enrollment in the study, MDR (Multidrug-resistant), RR (Rifampicin resistant, XDR (Extensively drug-resistant), I M (Isoniazid Mono resistance), ENRS (Electronic Nominal Record System).

Patients who were enrolled for the treatment before January 2014, transferred outpatients and those who were still under treatment on the final day of data collection, as well as resistance other than the most crucial drugs isoniazid and rifampicin were excluded from the study (Figure 1).

### 2.3. Variables Outcomes and Definitions

Treatment outcomes of the study participants were categorized according to the WHO definition, death, treatment failure and lost to follow-up were collectively grouped as “unsuccessful treatment outcomes” while cure and treatment completion were grouped as “successful treatment outcomes”. Based on starting of TB treatment after the onset of the symptoms, patients were classified into “Delayed” and “Not-Delayed,” taking 30 days (4 weeks) as cut-off points. All definitions are presented in Table 1 [14,15].

### 2.4. Identification and Antimicrobial Susceptibility Testing

All DR TB samples were collected under the supervision of qualified professionals in the TB control center. A sample was subjected to equal division into two parts, i.e., one portion of samples for the Xpert MTB/RIF assay (Cephid, Sunnyvale, CA, USA) and smear microscopy, and similarly, the second portion of samples was assigned for DST (drug-susceptible test) and Lowenstein–Jensen culture medium. Sputum samples from patients with positive Xpert MTB/RIF and Ziehl–Neelsen stain results were sent to the National Institute of Health Islamabad (NIH) for culture and DST analysis at the NIH laboratory. Drug-susceptible tests against RIF, ethambutol (EMB), isoniazid (INH), ofloxacin (OFX), capreomycin (CM), streptomycin (SM) kanamycin (KM), amikacin (AM), and ethionamide were performed utilising the agar proportion methods in medium on Middle Brook 7H10 as reported previously [16]. The concentrations include rifampicin (1 µg/mL), EMB (5 µg/mL), INH (0.2 µg/mL), OFX (2 µg/mL), SM (2 µg/mL), KM (5 µg/mL), ethionamide (5 µg/mL), CM (4 µg/mL), and AMK (4 µg/mL). Similarly, DST was carried out for pyrazinamide (PZA) by means of BACTEC 7H12 radiometric medium (Becton Dickinson, New Jersey, USA) following the manufacturer’s instructions. Furthermore, DST was made available for all the DR TB patients at registration time and repeated when considered essential. A sputum smear and culture were carried out based on a scheduled visit.

### 2.5. Treatment Protocol

Patients found to be resistant in Xpert MTB/RIF diagnostic test were registered as DR TB cases and treated with treatment regimen protocol in compliance with WHO and Pakistan national MDR TB control guidelines [9,15]. Before initiation of the regimen, baseline laboratory diagnostic tests were performed to determine complete blood count, hepatitis, HIV, blood sugar level, kidney, and liver function tests. All patients’ adherence was ensured by the pharmacist, doctors, treatment coordinators, and trained supporters. Patients were prior informed for every scheduled follow-up visit, and free laboratory tests and medications were provided at each follow-up visit to patients.

### 2.6. Statistical Analysis

Statistical Package for the Social Sciences, version 23 (SPSS^®^ IBM Corp., Chicago, IL, USA) was used for performing statistical analysis. Factors related to the treatment outcomes of drug-resistant tuberculosis were assessed using descriptive statistics (frequency, percentages) and logistic regression models. Multivariate binary logistic regression analysis was conducted to determine the final factors associated with unsuccessful outcomes statistically significant (*p* ≤ 0.05). Variables found significant with *p*-value (<0.15) in the univariate regression analysis were the criteria for addition in the final multivariate regression model [17,18]. In developing the multivariate binary logistic regression, we have checked the collinearity and tolerance value for all variables. If the variables had a high association with one another (Variance inflation factor = 10 and Tolerance value > 0.1), then one of them was removed from the concluding model [19]. Hosmere Lemeshow test was also applied for the adjustment of the final multivariate binary logistic regression model. Two different categories with binary variables were made for the treatment outcome, i.e., successful and unsuccessful. Odds ratios with 95% Confidence intervals with (*p* ≤ 0.05) were calculated to measure the level of association between variables and outcomes.

## 3. Results

During the study period, 308 DR TB patients were treated at the study site and 277 of them met the inclusion criteria and were analysed. The group of patients who were excluded from the study, included two poly-drug-resistant cases and 13 patients had left the study center. Similarly, the final treatment outcomes for 16 individuals were unknown because they were still on treatment. Out of the total 277, MDR/RR-TB cases were (265, 95.67%), isoniazid mono resistance cases were (8, 2.89), and XDR TB (4, 1.44%) (Figure 1). In most of the cases (47.3%), patients were between 18 and 40 years old. Patients from rural areas made up 66.8%, with males accounting for 55.2 percent of the total. About 52.7% of patients showed 30 days of delay before reporting to the MDR TB center and 9.4% of patients have already used second-line drugs and found resistance before being diagnosed with drug-resistant tuberculosis. Among the other patients, 17.7% had any comorbidity such as diabetes (26 patients), hypertension (11 patients), hepatitis (6 patients), and HIV (5 patients) (Table 2).

### 3.1. Drug Resistance

In this study, 84.5% of participants were previously treated for TB infection. Resistance to first-line drugs (FLD) was seen in almost all patients with at least one or more first-line drugs. Resistance to two major FLD was noted in 60 patients (21.6%), followed by resistance to four FLD drugs 18.4%, while 7.9% of patients were resistant to all five FLDs. Out of the total second-line resistant cases, i.e.,27.1%, 22.02% were resistant to at least any SLD fluoroquinolones (ofloxacin, levofloxacin, and moxifloxacin), followed by capreomycin, kanamycin, and amikacin (Table 3).

### 3.2. Predictors of Unsuccessful Treatment Outcomes

Treatment outcomes of the study participants (36.1%) were categorized according to the outcomes of death (21.7%), treatment failure (11.5%), and lost to follow-up (2.9%) and were collectively grouped as unsuccessful treatment outcomes. Cure (153, 55.2%), and treatment completed (24, 8.7%) were grouped as successful treatment outcomes (63.9%) (Table 4).

The logistic regression univariate analysis has shown that the following variables were significantly associated with unsuccessful treatment outcomes: gender, age above 60 years, and delay in reporting to drug-resistant tuberculosis center, comorbidities, and history of second-line drug resistance before being diagnosed with drug-resistant tuberculosis. In the multivariate analysis, only four major significant predictors of unsuccessful treatment were identified, such as male gender (AOR; 1.92, 95% CI 1.10–3.36), age group above 60 years (AOR; 3.34, 95% CI 1.09–10.1), comorbidities (AOR; 3.51, 95% CI 1.35–9.12), and history of second-line drugs resistance before being diagnosed with drug-resistant tuberculosis (AOR; 3.51, 95% CI 1.35–9.12) Table 5.

## 4. Discussion

This research showed the prevalence of predictors that impact DR TB treatment outcomes in Pakistan, a highly TB-endemic low-middle-income country. The treatment outcomes were briefly analyzed in accordance with the definitions put forward by the WHO [14,15]. In the current cohort, a total of 177 (63.9%) cases achieved successful treatment outcomes. Among these, 153 (55.2%) were declared cured, while 24 (8.7%) had completed their treatment. Of the remaining 100 (36.1%) patients with unsuccessful outcomes, 60 (21.7%) died, 32 (11.5%) were LTFU, and 8 (2.9%) were declared treatment failure. Thus, the site did not achieve the WHO recommended target of ≥75% for treatment success [18]. The treatment success rate (63.9%) observed in the current cohort was in line with the success rates reported by a meta-analysis (63.8%) [20], a study conducted in Sudan (63.5%) [21], and in China (63.4%) [22]. However, it was comparatively better than the treatment success rates reported in studies conducted in Morocco (53.5%) [23], Armenia (56.5%) [24], Ukraine (18.1%) [25], and India (38%) [26]. Differences in the study population in terms of age, gender, presence of comorbidities, disease severity, tobacco use, drug resistance pattern, social determinants of health, and socioeconomic characteristics could be some of the possible reasons for the discrepancy in treatment outcomes across these studies [13,27,28,29]. Another factor that may have contributed to the poor outcomes in the current study is the overburden of patients who were registered from different parts of the country in the TB care unit, which restricts the TB treatment coordinators access to the patients. The statement of the current study is supported by earlier studies that found overburdened healthcare staff in Pakistan TB centers [1,13]. Based on the results of this research, we recommend that early diagnosis, appropriate therapy, regular supportive care, and health advocates programmes should be implemented for patients who are at risk of poor outcomes. This might be possible by giving awareness about district and provincial drug-resistant tuberculosis centers to all communities and health care centers to register patients at the nearest DR TB control center.

In the present study, the prevalence of death rate was 21.7%; this figure was similar to reports from previous studies conducted in Colombia, India, and South Africa [30,31,32]. However, this was lower than the death rate reported in studies from Western India and Ukraine [25,26]. The higher death rate in the current study may be due to the delayed diagnosis and low education regarding DR TB, disease severity, comorbidities, and previously TB treated cases [24,25]. In the current study, the failure rate of 2.8% was lower than the failure rates reported in previous studies conducted in Ethiopia 12.8% [33] and Armenia 14.3% [24]. This might be due to a regular supply of drugs and counselling by the pharmacist and psychiatrist, regular checkups by the medical officer, and scheduled monthly visits by data coordinators. Lost to follow-up from TB treatment health centers is one of the main challenges for TB control programs. In the current study, overall, 11.5% of patients were LTFU. Previous studies conducted in Morocco 34.6% [23], Ukraine 31.9% [25], South Africa 20.9% [32], and Ethiopia 9.7% [33] showed more than 11.5% of patients were LTFU. The difference in the percentage of LTFU rates among studies may be due to regular home visits by the treatment coordinator, the presence of qualified doctors for follow-up checkups, proper social support by a psychiatrist, and the provision of free medicine with proper counselling by the pharmacist. Despite free therapy, psychologist and pharmacist counseling, and home coordinator visits, our study’s LTFU rate is also a point of concern for the management of DR TB. Perhaps it indicates a need for better access to more effective, less toxic, and easier to implement drug regimens, along with proper engagement of patients in the treatment plan. The current study LTFU rate may be associated with comorbidities, resistance to SLD, previous history of pulmonary TB treatment, gender, and deaths that were not reported to the TB center and access to the PMDT site [34,35].

The multivariate analysis showed numerous other factors that played an essential role in poor treatment outcomes including gender (male), age (above 60), history of past used SLD, and comorbidities. In this study, male participants have a more likely chance of poor treatment outcomes than females. The findings are consistent with previous studies [36,37], while other studies explained an opposite statement [38,39]. This difference between the reports might be due to financial requirements, illiteracy rate, exposure to environmental air-pollution, tobacco use, and fear of stigmatization which make it more difficult for patients in less developed countries to achieve therapeutic goals [40,41,42]. Therefore, community surveys and randomized controlled trials need to be conducted on gender discrepancies and socioeconomics parameters at the national level. The other significant predictor that we assessed was the age group of more than 50 years, which had 3.34 times the risk to develop a poor treatment outcome. Older age has previously been studied as a significant factor associated with poor treatment outcome [43,44]. The reason behind this may be the physical weakness, daily complex medications routine, follow-up visits, malnourishment, comorbidities, and weak immunity. All these factors together increase the possibility of older age patients being more towards poor outcomes [20].

In the present study, comorbidities were also found associated with an increased relative risk of poor treatment outcome in DR TB patients. This study result is in concordance with a meta-analysis [20] and study conducted in Brazil and Yemen [45,46]. This current finding will allow and help policymakers to develop new care strategies with more focus on early detection and patient-centered care during comorbid conditions. Patient-centered care has been described as an essential predicator for positive outcomes in DR TB [47].

The odds ratio of poor treatment outcomes was high among those patients who had already used any SLD and found resistance before proper DR TB treatment. The results of this study are similar in comparison to other studies [45,48]. An approximate 21.02% of cases were subject to any form of fluoroquinolone resistance. The fluoroquinolone resistance rate is higher in this study, while the observed rate of fluoroquinolone resistance is similar to other studies conducted in Pakistan [5,49]. Such a high proportion of fluoroquinolone resistance could be related to the non-prescription sale of antibiotics, delay in diagnosis, easy access of patients to antibiotics, non-formal health care practices, and irrational prescriptions [50].

The current study was conducted according to standardized WHO procedures, but it has several limitations. First of all, this study was carried out in a single center where patients were registered from most parts of the country. Secondly, because of the retrospective nature of this study, numerous significant clinical characteristics that may have influenced an unsuccessful treatment outcome were not observed and evaluated. Therefore, future prospective interventional studies should be based on patient-related, drug-related, and health system-related factors, to find out compact national decisions for successful treatment outcomes.

## 5. Conclusions

Our study showed a treatment success rate of 63.9% among DR TB patients, and we conclude that the successful treatment outcome was lower than the success rate set by WHO (≥75%). Gender (male), age above 60, history of past SLD use, and comorbidities were found to be significantly associated with poor treatment outcomes. All of these variables show a need for the development of unique and innovative strategies to monitor and evaluate DR tuberculosis patients. These findings demonstrate how serious DR TB is in this region and we recommend a strong systematic approach to decrease the number of deaths and default rates through proper management and long-term interventions.

## Figures and Tables

**Table 1:** Category of treatment outcome, type of TB resistance, and previous history of TB patients registered modified from WHO definitions, comorbidities.

Treatment Outcomes	Definition
Cured	A patient who has completed the treatment as recommended by the national policy, three consecutive smear-negative cultures in months taken at least one month apart after the intensive phase
Treatment completed	According to national policy, a patient who has accomplished the time of management but has no evidence of failure due to any reason with no record
Successful outcomes	“The sum of Cured and Treatment completed”
Treatment Failure	A patient whose treatment plan needed to be terminated or changed to new treatment plan due to no clinical response, adverse drug reactions or treatment resistance
Lost to follow-up	A patient whose management was disturbed for ≥ 2 months after registration
Died	Registered in the medical record as died before or after starting the course of treatment
Unsuccessful outcomes	Died + Lost to follow up + Treatment failure
Types of Resistance	
Drug resistance TB	Mycobacterium tuberculosis strains showed resistance to at least one anti TB drugs
Mono resistance TB	Resistance to any single first-line anti-TB drug (isoniazid, rifampicin, ethambutol, or pyrazinamide)
Poly drug resistance TB	Resistance to more than one first-line anti-TB drug, other than most important drugs isoniazid and rifampicin
Multidrug resistance TB (MDR)	TB strains resistant to at least both common drugs isoniazid and rifampicin
Rifampicin resistance TB (RR)	It includes any resistance to rifampicin, in the form of mono-resistance, poly-resistance, MDR, or XDR, and RR-TB cases are often grouped together as MDR/RR-TB
Isoniazid-resistant TB	TB strains resistance to isoniazid and susceptible to rifampicin
Extensive drug resistance (XDR)	Resistance to any fluoroquinolone, and at least one out of three second-line injectable drugs (capreomycin, kanamycin, and amikacin), in addition to MDR TB resistance
Patient type on basis of the history of TB	
Previously treated	“Previously treated refers to patients who have received 1 month or more of anti-TB medicines in the past”
New Patient	The new case is defined who has taken anti-TB medicines for less than 1 month.
Delays to diagnosis and treatment	
Delayed	Delayed” taking treatment after 4 weeks/30 days after the onset of MDR TB symptoms
Not Delayed	Not Delayed” taking treatment within 4 Weeks/30 days after the onset of MDR TB symptoms

**Table 2:** Sociodemographic and clinical characteristics of MDR TB patients (*n* = 277).

Characteristics	Patients, N (%)
Marital status	
Married	224 (80.9)
Unmarried	53 (19.1)
Gender	
Male	153 (55.2)
Female	124 (44.8)
Age	
≤20	49 (17.7)
21–40	131 (47.3)
41–60	77 (27.8)
>60	20 (7.2)
Employment►	
Employed	29 (10.5)
Unemployed	70 (45.5)
Student	46 (16.6)
House Wife	77 (27.4)
Province	
Punjab	135 (48.7)
Khyber-Pakhtunkhwa	25 (9.0)
Federal Islamabad	73 (26.4)
Azad Jammu Kashmir	44 (15.9)
Distance from health care center	
0–10 Km	38 (13.7)
11–20 Km	30 (10.8)
21–30 Km	46 (16.6)
>30 Km	163 (58.8)
Residency	
Rural	185 (66.8)
Urban	92 (33.2)
Baseline Weight (kg)	
<40	64 (23.1)
≥40	213 (76.9)
Reported to MDR center	
Within 30 days	131 (47.3)
After 30 days	146 (52.7)
Comorbidities	
Diabetes	26 (9.3)
Hypertension	11 (3.9)
Hepatitis	6 (2.1)
HIV	5 (1.8)
Parkinson disease	1 (0.3)
Sputum smear	
Negative/Scanty	106 (38.3)
Positive	171 (61.7)
Previous TB treated	
Previously TB treated case	234 (84.5)
New Case	43 (15.5)
History of SLD resistance	
Yes	26 (9.4)
No	251(90.6)
Resistance to any SLD drugs	
No, resistance to any SLD	202 (72.9)
Yes, resistance to SLD	75 (27.1)

FLD (First line of drugs); SLD (Second line of drugs) ► Data available only for 221 patients.

**Table 3:** Patterns of drug resistance among drug-resistant tuberculosis patients (*n* = 277).

Number of Drugs	N (277)	100%
FLD resistance		
Rifampicin resistance	54	31.0
Isoniazid resistance	8	2.8
Isoniazid+ Rifampicin resistance	60	21.6
Isoniazid+ Rifampicin+ Streptomycin resistance	15	5.4
Isoniazid+ Rifampicin+ Pyrazinamide resistance	21	7.5
Isoniazid+ Rifampicin+ Ethambutol resistance	10	3.6
Isoniazid+ Rifampicin+ Ethambutol+ Streptomycin resistance	23	8.3
Isoniazid+ Rifampicin+ Pyrazinamide+ Ethambutol resistance	20	7.2
Isoniazid+ Rifampicin+ Pyrazinamide+ Streptomycin resistance	8	2.8
Isoniazid+ Rifampicin+ Ethambutol+ Streptomycin+ Pyrazinamide resistance	22	7.9
FLD resistance to several Drugs		
2 FLD Drugs resistance	60	20.5
3 FLD Drugs resistance	46	16.6
4 FLD Drugs resistance	51	18.4
5 FLD Drugs resistance	22	7.9
SLD resistance		
Fluoroquinolones alone resistance (ofloxacin, levofloxacin, moxifloxacin)	61	22.02
Resistance to Amikacin alone	2	0.7
Resistance to Capreomycin alone	2	0.7
Resistance to Kanamycin alone	1	0.3
SLD resistance to several Drugs		
Resistance to Capreomycin+ Moxifloxacin	3	2.5
Resistance to Amikacin + Levofloxacin	1	0.3
Resistance to Amikacin + Kanamycin	2	0.7

FLD (first line of drugs); SLD (second line of drugs).

**Table 4:** Trends of TB treatment outcome audit of 6 years among TB patients (*n* = 277).

TB Outcomes/Year n (%)	2014	2015	2016	2017	2018	2019-J *	Total
Cured	29	38	26	27	28	5	153 (55.2)
Completed Treatment	0	5	5	6	5	3	24 (8.7)
Successful outcomes	29	43	31	33	33	8	177 (63.9)
Failure	3	3	0	0	1	1	8 (2.9)
Lost to Follow Up	3	5	6	9	5	4	32 (11.5)
Died	13	10	7	7	13	10	60 (21.7)
Unsuccessful outcomes	19	18	13	16	19	15	100 (36.1)

2019-J * means until July 2019 cases were included in the analysis. Treatment outcomes were categorized according to WHO recommendations.

**Table 5:** Univariate and multivariable logistic regression for successful and unsuccessful predictor related treatment outcomes among patients (*n* = 277).

Predictor	Successful Outcomes	Unsuccessful Outcomes	COR 95% CI	AOR 95% CI
Marital status				
Married	144 (81.4)	80 (80)	Referent	Not included
Unmarried	33 (18.6)	20 (20)	1.09 (0.58–2.02)	
Gender				
Female	92 (51.4)	32 (32.7)	Referent	Referent
Male	87 (48.6)	66 (67.3)	2.00 (1.20–3.37) *	1.92 (1.10–3.36) *
Age				
>20	33 (18.6)	18 (18)	Referent	Referent
21–40	(57.1)	33 (33)	0.59 (0.29–1.20)	0.44 (0.21–0.93)
41–60	36 (20.3)	34 (34)	1.73 (0.82–3.63)	1.39 (0.64–3.02)
>60	7 (4)	15 (15)	3.92 (1.35–11.3)	3.34 (1.09–10.1) *
Employment►				
Unemployed	41 (23.1)	29 (29)	Referent	Referent
Employed	20 (11.3)	9 (9)	1.61 (0.68–3.80)	Not included
Student	35 (26.6)	11 (11)	1.64 (0.67–4.04) *	
House Wife	46 (19.8)	31 (31)	0.76 (0.27–2.15)	
Province				
Punjab	91 (51.4)	44 (44)	Referent	Not included
Khyber Pakhtunkhwa	15 (8.5)	10 (10)	1.37 (0.57–3.31)	
Federal Islamabad	46 (26)	27 (27)	1.21 (0.66–2.20)	
Azad Jammu Kashmir	25 (14.1)	19 (19)	1.57 (0.78–3.15)	
Distance from health care center				
0–10 Km	27 (15.3)	11 (11)	Referent	Not included
11–20 Km	21 (11.9)	9 (9)	1.05 (0.36–3.00)	
21–30 Km	31 (17.50	15 (15)	1.18 (0.46–3.02)	
>30 Km	98 (55.4)	65 (65)	1.62 (0.75–3.50)	
Residency				
Rural	120 (67.8)	65 (65)	Referent	Not included
Urban	57 (32.2)	35 (35)	1.13 (0.67–1.90)	
Baseline Weight (kg)				
<40	42 (23.7)	22 (22)	Referent	Not included
≥40	135 (76.3)	77 (78)	1.10 (0.61–1.98)	
Reported to MDR center				
Within 30 days	102 (57.6)	44 (44)	Referent	Referent
After 30 days	75 (42.4)	56 (56)	1.73 (1.05–2.83) *	1.57 (0.91–2.71)
Comorbidities				
Without comorbidities	154 (87)	74 (74)	Referent	
With comorbidities	23 (13)	26 (26)	2.35 (1.25–4.39) *	2.69 (1.35–5.38) **
Sputum smear				
Negative/Scanty	67 (37.9)	39 (39)	Referent	Not included
Positive	110 (62.1)	61 (61)	1.05 (0.63–1.73)	
Previous TB treated				
New Case	24 (13.6)	19 (19)	Referent	Not included
Previously treated case)	153 (86.4)	81 (81)	1.49 (0.77–2.89)	
History of SLD resistance				
No	171 (95.5)	82 (80)	Referent	
Yes	8 (4.5)	18 (20)	4.63 (1.93–11.1) *	3.51 (1.35–9.12) *
Resistance to any SLD drugs				
No resistance to any SLD	130 (73.4)	72 (72)	Referent	Not included
Yes resistance to SLD	47 (26.6)	28 (28)	1.07 (0.62–1.86)	

SLD (Second Line Drug), Reference Category, (Unsuccessful outcomes), COR (crude odds ratio), AOR (adjusted odds ratio), CI (confidence interval), (Univariate analysis; *p* < 0.15 is considered significant), Multivariate model was significant, with chi square model coefficients 47.6 = (DF 7, *N* = 277), *p* <0.0005, Hosmer–Lemeshow statistic chi square = 7.54 (DF = 8, *N* = 277), *p* = 0.47, Collinearity (Variance inflation factor = 10), Tolerance value <0.1. * *p* > 0.05, ** *p* > 0.001, ► missing data; reason why not included in final analysis.

## Data Availability

The data sets used and analyzed during the current study are available from the corresponding author on reasonable request.
